# Identifying targeted cell-free DNA methylation regions in head and neck cancer via paired methylome analysis

**DOI:** 10.21203/rs.3.rs-5124805/v1

**Published:** 2024-11-26

**Authors:** Tingyi Li, Krupal B. Patel, Xiaoqing Yu, Sijie Yao, Liang Wang, Christine H. Chung, Xuefeng Wang

**Affiliations:** H Lee Moffitt Cancer Center and Research Center Inc: Moffitt Cancer Center; City of Hope Comprehensive Cancer Center: City of Hope Inc; H Lee Moffitt Cancer Center and Research Center Inc: Moffitt Cancer Center; H Lee Moffitt Cancer Center and Research Center Inc: Moffitt Cancer Center; H Lee Moffitt Cancer Center and Research Center Inc: Moffitt Cancer Center; H Lee Moffitt Cancer Center and Research Center Inc: Moffitt Cancer Center; H Lee Moffitt Cancer Center and Research Center Inc: Moffitt Cancer Center

## Abstract

Despite the critical need, progress in developing cell-free DNA (cfDNA) liquid biopsy biomarkers for the diagnosis and risk stratification of head and neck squamous cell carcinoma (HNSC) has been limited. In this study, we present a comprehensive paired-sample differential methylation region (psDMR) analysis in HNSC, aimed at identifying reliable and HNSC-specific regions for cfDNA biomarker discovery. Traditional DMR analyses often overlook paired-sample information and fail to account for the heterogeneity within HNSC tissues. Our findings reveal a substantial overlap of hypermethylated DMRs across two independent HNSC methylation datasets, demonstrating the robustness and clinical relevance of these regions for cfDNA biomarker development. Furthermore, we identified consistent DMRs in both HNSC and lung squamous cell carcinoma (LSCC), suggesting the potential for pan-squamous cell carcinoma biomarkers. Notably, gene clusters such as *HOXD*, *ZNF*, and *NKX2* were frequently hypermethylated, providing new insights into the shared epigenetic landscape of HNSC and LSCC. This study underscores the importance of incorporating matched normal tissues in cancer methylome analyses and establishes a foundation for advancing cfDNA-based biomarker discovery across squamous cell carcinomas.

## INTRODUCTION

In recent years, the field of cancer research has witnessed a promising advancement with the emergence of cell-free DNA (cfDNA) analysis, which has opened new avenues for non-invasive assessment of tumor-derived genetic and epigenetic alterations. Liquid biopsies from cancer patients, such as plasma, urine, and saliva, serve as reservoirs for cfDNA that carry fragments of circulating tumor DNA (ctDNA), which are shed into circulation because of cancer cell death and secretion. cfDNA found in health or cured individuals predominantly originates from hematopoietic cells, specifically lymphocytes and erythrocyte progenitors. Therefore, the cfDNA obtained from individuals belonging to different clinical groups, including those with varying tumor burden, stage, or treatment regimens, as well as patients throughout their treatment journey, may exhibit considerable variations in both concentration and composition. In general, cfDNA methylation exhibits greater sensitivity in detecting early-stage cancer due to extensive epigenetic alternations occurring during in the initial phases of cancer progression, as opposed to mutation-based methods^1^. By leveraging the distinct methylation signatures found in cfDNA, clinicians and researchers can gain valuable insights into tumor progression, response to treatment, and even the emergence of treatment-resistant clones. Moreover, the non-invasive nature of cfDNA analysis allows for repeated testing and real-time monitoring of disease dynamics, offering a minimally invasive approach that circumvents the limitations and risks associated with traditional tissue biopsies. These advancements thus paved the way for personalized medicine, where tailored treatment strategies can be developed based on the unique cfDNA methylation profiles of individual patients.

There are many commonly accepted cfDNA methylation biomarkers that have been identified and validated across various types of cancer. For example, the hypermethylation of gene *SEPTIN9* (*SEPT9*) has been extensively studied as an early screening biomarker for colorectal cancer^2^; methylation status of *SHOX2* has been utilized in both lung cancer^3,4^ and colon cancer^5^ detection; *MGMT* has been studied in relation to brain tumors^6^; *RASSF1A* has been shown as a potential candidate for pan-cancer biomarker^7,8^, aiding both cancer diagnosis and prognosis^9^. Emerging evidence also indicates that the methylation patterns of long interspersed nuclear element (LINE-1) hold promise as a new global cfDNA biomarker for cancer hallmark in multiple cancers^10,11^. Considering the intrinsic heterogeneity of cancer and the tissue-specific nature of DNA methylation, it is anticipated that the inclusion of multiple biomarkers in a predictive model will enhance both the sensitivity and specificity of cancer detection. Therefore, a panel of cfDNA methylation biomarkers should be tailored for each cancer type or even subtype, ideally also taking into account the technical biases and preferences associated with the specific cfDNA methylation profiling technologies being utilized.

Despite the capability of modern genome-wide cfDNA methylation assays to provide a holistic assessment of sites in a single experiment, it is important to note that many successful studies in cfDNA continue to rely heavily on candidate biomarkers derived from the tumor tissue data. In our opinion, this is attributed to three main reasons: Firstly, the relatively small sample size and the temporal heterogeneity in current cfDNA studies pose limitations on effective training; Secondly, the differential methylation analysis by comparing methylome of tumor and normal tissues remains a powerful approach for identifying cancer-specific biomarkers; Thirdly, given the low amount of cfDNA in liquid biopsies, the selective enrichment of cfDNA fragments over the background non-cancer-specific regions will greatly increase the detection sensitivity in most techniques. Our literature review indicates the methylation data generated from The Cancer Genome Atlas (TCGA) project remain the mainstay for the initial methylation region discovery in most cancers. For example, in a study for discovering non-invasive blood-based markers for lung cancer^12^, a panel of 12,899 pre-selected cancer-specific informative regions was first constructed based on TCGA lung cancer database.

Due to the initial emphasis of the TCGA project on investigating the landscape of cancer genomes, the methylation data in TCGA has predominantly been obtained from tumor tissues. For example, within the TCGA Head-Neck Squamous Cell Carcinoma (TCGA-HNSC) dataset, there are 528 tumor tissues available for methylation data, whereas only 50 normal tissue samples were profiled with methylation. In our previous study^13^, we discovered that the more significant differential methylated regions (DMRs) can be detected by including only those samples with paired tumor and normal tissues in the analysis. This finding underscores the critical importance of conducting paired-sample differential methylation analysis (psDMR), as it enhances the statistical power and reliability of identifying candidate regions. In the context of cancers such as HNSC, which exhibit remarkable sample heterogeneity due to tumor site and molecular subtypes, the self-controlled psDMR becomes even more crucial. By comparing methylation patterns within paired samples, we can effectively account for the inherent variability and confounding factors associated with such heterogeneity, leading to more accurate and insights into the epigenetic dynamics of HNSC.

In this study, our main objective is to conduct a thorough psDMR analysis of the methylation data in HNSC with the aim of developing an enhanced resource in the form of methylation panel that can be effectively utilized in targeted cfDNA methylation studies. To achieve this, we embark on a systematic approach. We first revisit the TCGA data and conduct psDMR analysis based on the updated HumanMethylation450 (450K) methylation data sourced from HNSC-TCGA, which has been reprocessed using the SeSAMe pipeline^14^, ensuring refined data quality. Our main analysis is based on the Infinium MethylationEPIC DNA methylation (EPIC array) data obtained from matched tumor-normal samples in the CPTAC-HNSC study^15^. By conducting a similar psDMR analysis on two independent datasets, we aim to validate our findings from the original TCGA data and identify additional methylation sites of interest. Lastly, we demonstrate the application of the targeted psDMR regions based on two cfDNA studies. These studies provide real-world examples of how the identified psDMR regions can be effectively employed in investigating cfDNA methylation patterns for cancer detection, especially in scenarios with limited sample sizes. Importantly, the comprehensive biomarker prioritization strategy and findings presented in this study have the potential to extend beyond HNSC, as they can be applied to the analysis of methylation data in other cancer types. Collectively, by demonstrating the practical utility and effectiveness of the targeted psDMR analysis, we contribute to the advancement of cfDNA-based approaches for early cancer detection and monitoring, broadening the scope of their impact in the field of cancer research.

## METHODS

### Bulk tumor and liquid biopsy methylation data

The EPIC array data generated from the Clinical Proteomics Tumor Analysis Consortium (CPTAC)-HNSC, and CPTAC-LUSC cohorts and the Infinium HumanMethylation450 (450K array) data generated from The Cancer Genome Atlas (TCGA)-HNSC cohort, as well as the associated clinical data, were downloaded from the Genomic Data Commons (GDC) portal (https://portal.gdc.cancer.gov/). For consistency across our analyses, we used the normalized methylation levels (beta values) processed using a new bioinformatics methylation pipeline called the Sensible Step-wise Analysis of Methylation data (SeSAMe) pipeline. This report also represents the first comparison analysis of methylation data from CPTAC and TCGA, utilizing the latest SeSAMe-processed data uploaded to the GDC portal. In total, we identified 42 paired tumor and normal tissue samples in the CPTAC-HNSC cohort and 50 pairs of samples in the TCGA-HNSC cohort. In the CPTAC lung cancer dataset, we identified 92 paired tumor and normal tissues from patients with lung squamous cell carcinoma (LSCC). The Moffitt validation dataset, consisting of pre- and post-surgery plasma samples from patients with oral cavity squamous cell carcinoma (OCSCC), was previously detailed^13^. This data was generated based on the hypothesis that the methylation pattern of cfDNA would rapidly converge to background levels following surgery, and that the DMR analysis would reveal OCSCC-specific biomarkers. The additional validation dataset, comprising plasma-based cfDNA methylation profiles from various cancer types including nasopharyngeal carcinoma, was downloaded from the Gene Expression Omnibus (GEO) with the accession number GSE124775. Generated based on a novel multiplex 5-methylcytosine marker barcode counting (MMBC-seq) technique, this validation dataset contains the cell-free DNA methylation profile from 91 targeted genes from 121 cancer patients and 58 healthy individuals. To test cancer specificity of biomarkers, we classified all samples into three groups: nasopharyngeal carcinoma, other malignancies, and healthy.

### Paired sample DMR analysis

The psDMR analyses comparing bulk tumor and matched normal tissues DNA methylation profiles were performed using the champ.PairedDMR function implemented in the “ChAMP” R package with default parameter settings. This includes a minimum cluster size at seven probes and the selection of array types (EPIC and 450K). The example code for this analysis is as follows: *dmr_results<- champ.PairedDMR(beta = beta_matrix, pair = mypair, pheno = mypheno, cores = 3, minProbes = 7, arraytype=“EPIC”)*. As a comparison, the unpaired (bumphunter) DMR analysis can be performed using the same package as follows: *dmr_results.unpaired<- champ.DMR(beta = beta_matrix_impute$beta, pheno = mypheno, method=“Bumphunter”, cores = 3, minProbes = 7, arraytype=“EPIC”)*. The PairedDMR method first clusters adjacent CpGs into regions, typically comprising several CpGs. For each CpGs, a paired t-test is employed to compare paired samples between two distinct phenotypes. The t value for each CpG serves as an indicator of whether it presents a paired differential methylated probe (paired-DMP). To mitigate the impact of outliers, the loess function is applied to the t values of all CpGs within a region. To assess the statistical significance of each CpG region, the function performs sampling throughout the entire genome to simulate regions containing a similar count of random CpGs. By aggregating the smoothed t value of these simulated regions, null distribution is established, from which the p-value for the specific CpG region is derived. This function returns candidate DMRs with information including value, area, cluster, and Family-Wise Error Rate (FWER) adjusted p-value similar to the output format utilized by the DMR function in the “bumphunter” R package. The identified DMRs were subsequently annotated with respect to gene location using the annotateDMRInfo function from the “methyAnalysis” R package. DMRs located in promoter regions and exhibiting a FWER less than 0.05 were prioritized in the downstream comparative analysis.

### Summary report of DMR results

Our main biomarker discovery was informed by the CPTAC-HNSC analysis, as this study represents the first psDMR analysis on the EPIC array in head and neck cancer. The top candidates that were located in gene regions previously established as cfDNA or liquid biopsy methylation biomarkers received further attention in our report. The comparison between the results of the CPTAC-HNSC and TCGA-HNSC datasets was conducted based on the extent to which top DMRs from one dataset could be recapitulated in the other. Additional emphasis was given to the DMRs that were consistently identified in both datasets. The analysis of DMRs in cfDNA methylation data has been previously described^13^. Briefly, we used the “MEDIPS” R package to calculate normalized methylation levels across all candidate ROI (region of interest) regions identified in the tumor-normal psDMR analysis. The differential analysis for each genomic window was then conducted using the fitNBglm function from the “qsea” R package.

## RESULTS

### psDMR on CPTAC-HNSC reveals candidate regions for cfDNA biomarkers

The psDMR analysis conducted on the methylation data obtained from 42 paired tumor and normal tissues in CPTAC-HNSC cohort successfully identified a total of 769 significantly differentially methylated regions with FWER < 0.05 (using the modified bumphunter algorithm implemented in *ChAMP)*. Out of these 769 regions, 573 were found to be located in promoter regions, corresponding to 561 unique genes. Within this gene set, it was observed that 11 genes, such as *PAX6*, *WT1*, and *CA10*, harbored multiple significant DMRs. The detailed DMR information regarding all significant DMRs are listed in **Supplementary Table 1**, while the top 30 DMRs and their associated names are highlighted in Table 1. Notably, among the top 30 DMRs, as well as among all significantly differentially methylated regions, a significant majority displayed hypermethylation when comparing tumor vs. normal tissues. Specifically, 83.3% (n = 25) of the regions in the top 30 DMRs and 73.1% (n = 562) of all significant DMRs exhibited hypermethylation.

In the top 30 DMRs, we observed that 14 are located within genes previously established as liquid biopsy methylation biomarkers in at least one cancer type to date, indicating the relevance and importance of these genes as reliable cancer-specific markers. These genes include *CALCA*, *ALX4*, *BOLL*, *ZIC1*, *ADCYAP1*, *ZNF577*, *NKX2-6*, *USP44*, *PCDHGA12*, *HOXD10*, *EYA4*, *ZNF781*, *HOXD9*, and *ZNF154*. The gene *CALCA*, encoding the calcitonin gene-related peptide that possess tumor suppressive potential, has been documented as being hypermethylated in several malignancies including head and neck cancer^16^. *CALCA* has also been reported as an effective cfDNA methylation biomarkers for early diagnosis of thyroid cancer^17^, ovarian cancer^18^, and testicular germ cell tumor^19^. The genes *ALX4* and *ZIC1* are transcription factors that play pivotal roles in regulation of embryonic development, and they are identified as epigenetically downregulated tumor suppressor^20,21^. The genes *HOXD9* and *HOXD10* belong to HOX gene cluster, and the methylation status of this gene family have been extensively explored as liquid biomarker in multiple cancers^22^. Notably, in our previous study we also reported the diagnostic potential of these two genes using plasma samples from OCSCC^13^. Other HOX genes identified from the significant DMRs include *HOXA2*, *HOXA3*, *HOXA7*, *HOXA9*, *HOXB1*, *HOXC4*, *HOXC6*, *HOXC9*, *HOXD4*, and *HOXD12*. Another noteworthy discovery that strongly aligns with previous analysis in OCSCC is the identification of ZNF family genes (e.g., *ZNF577*, *ZNF781*, *ZNF418 and ZNF154*) in the top-ranked list. The entire significant DMR list encompasses a total of 46 ZNF family genes. These genes are recognized for their roles in transcriptional regulation and tumor suppressor activity and have gained increased attention in the context of head and neck cancers^13,23^.

Another intriguing observation within the top 30 DMR list is the presence of three hypomethylated region (out of five) derived from microRNA genes, specifically *MIR411*, *MIR409*, and *MIR380*. Furthermore, a total of 21 significant DMRs were identified in microRNAs. Among them, additional microRNAs with hypomethylation status include *MIR381*, *MIR376C*, *MIR668*, *MIR127*, *MIR495*, *MIR124-3*, *MIR10B*, *MIR382*, and *MIR496*.

### Consistent DMRs validated by TCGA-HNSC psDMR analysis

We performed the similar analysis of the TCGA data by conducting psDMR analysis on the 450K array methylation data obtained from the paired samples in HNSC-TCGA. The beta-values (downloaded from GDC Data Portal) were processed via the same SeSAMe pipeline utilized in CPTAC, thus minimizing data inconsistency arising from different bioinformatics pipelines. The psDMR analysis on the TCGA data identified a total of 521 significantly differentially methylated regions with FWER < 0.05. Among them, 359 regions found to be located within gene promoter regions, corresponding to 355 unique genes. The top 30 DMRs from the TCGA analysis and their associated names are listed in **Supplementary Table 2.** Out of those DMRs, 29 regions (excluding *C11orf21*) and a notable majority of all significant DMRs, specifically 90.3% (324/359), displayed hypermethylation. The overall results demonstrated a high level of consistency between the findings from the CPTAC and TCGA. As indicated in the gene symbol column of the table, 28 out of top 30 DMRs in TCGA were also found significant in CPTAC. Even more strikingly, among these, a total of 12 DMR genes were consistently identified as top 30 DMRs in both the TCGA and CPTAC analyses. These genes are *ALX4*, *CALCA*, *ADCYAP1*, *PCDHGA12*, *C11orf21*, *ZIC1*, *ABCC9*, *EVX1*, *GFRA1*, *PHYHIPL*, *HOXD9*, and *NKX2-6*. Additionally, another 10 DMR genes were identified as top 100 DMRs in the CPTAC analysis, namely *EDNRB*, *CCNA1*, *ZIM2*, *MARCHF11*, *PENK*, *MIR124-2*, *ITGA8*, *ASB3*, *ZNF542P*, and *ZNF454*. By specifically examining DMRs located in promoter regions, we identified a notable overlap of 49 overlapped genes among the top 100 DMRs from two datasets and a total of 264 overlapped genes from the lists of all significant DMRs. This remarkable overlap highlights the robustness and reproducibility of psDMR results across independent datasets, providing further support for their potential translational significance in the context of HNSC liquid biopsy studies. Furthermore, the analysis revealed a significant presence of HOX and ZNF family genes in both TCGA and CPTAC datasets. Alongside HOXD9, which appeared in the top 30 list from the TCGA analysis, several other HOX genes including *HOXD10*, *HOXD12*, *HOXA7*, *HOXA9*, *HOXB1*, *HOXC4*, and *HOXC6* were also identified. Apart from *ZNF542P* and *ZNF454*, an additional set of 34 ZNF genes were found to harbor significant DMRs in the TCGA analysis. A total of 11 microRNAs were identified in the significant DMR analysis with TCGA data. Five of them displayed hypomethylation status; and four out of these five, namely *MIR411*, *MIR495*, *MIR496*, and *MIR668*, were also identified in CPTAC data.

**Figure 1:**
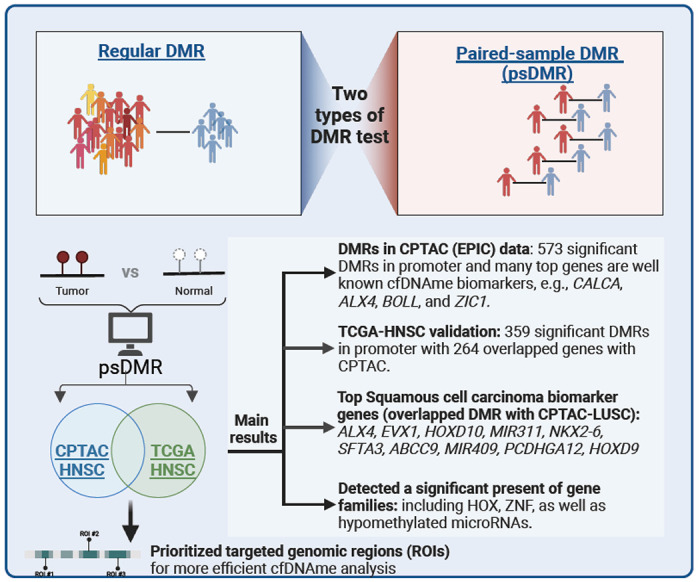
Workflow of paired sample DMR analysis and key findings in HNSC samples.

As summarized in Fig. 1, the comparative results of our study not only demonstrate the reliability of psDMR analysis across datasets but also contribute to expanding our understanding of the epigenetic landscape specific to HNSC.

### Leveraging targeted regions in genome-wide cfDNA DMR analysis

**Figure 2:**
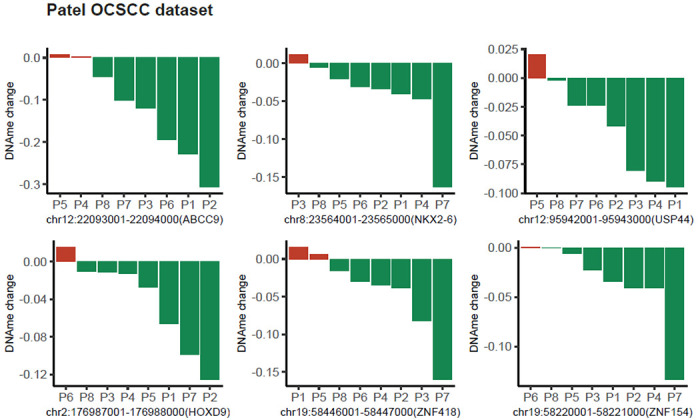
Validation of the top DMRs based on plasma cfDNA methylation data obtained the Patel OCSCC plasma liquid biopsy study.

In order to showcase the effectiveness of targeted regions in providing insights for liquid biopsy analysis in HNSC, we conducted a candidate region-based DMR analysis based on plasma cfMBD-seq obtained from eight patients diagnosed with OCSCC^13^. We performed genome-wide differential coverage analysis on cfDNA methylation data by only considering those regions determined to be significant in the comparison of matched tumor-normal tissues from the CTPAC analysis. The overall analysis steps of DMR on cfDNA methylation data followed a similar approach to our previous analysis. Specifically, we employed “MEDIPS” to calculate normalized methylation levels in all targeted ROI regions. Subsequently, fitNBglm function from the “qsea” package was utilized to perform differential analysis for each genomic window. Due to the restricted sample size and the validation-focused nature of the study, we employed unadjusted p-values as a criterion for prioritizing the top DMRs. Out of the analyzed regions, a total of 185 regions demonstrated statistical significance (P < 0.05). The top five DMR regions from the cfDNA data are identified within the promoter regions of the following genes: *PENK, TRH, PIEZO2, CA8*, and *HPSE2*. Remarkably, among the top 30 DMR genes identified based on the CPTAC psDMR analysis, we observed that six of them, namely *ABCC9, NKX2-6, USP44, HOXD9, ZNF418*and *ZNF154*, are also present in the significant DMR list obtained from the cfDNA targeted DMR analysis. The consistency observed in the overall pattern across patients, as depicted in Fig. 2’s waterfall plot, further reinforces the encouraging nature of these results in alignment with our previous analysis.

**Figure 3:**
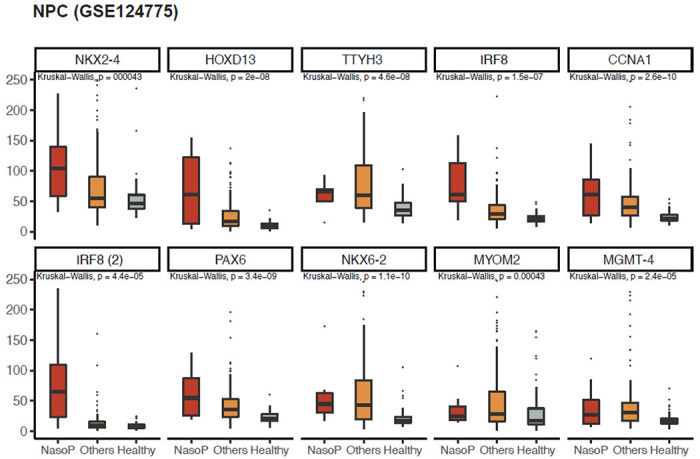
Validation of the top DMRs based on plasma cfDNA methylation data obtained from the NPC liquid biopsy study (GSE124775).

In another plasma-based cfDNA methylation from multiple cancer types including nasopharyngeal carcinoma (GSE124775), we found 17 genes (out of 91 targeted genes available in the dataset) overlapped with DMR genes identified based on the CPTAC psDMR analysis. The shared genes are *CCNA1, NKX2-4, FOXG1, PAX6, CNR1, NKX6-2, TWIST1, MYOM2, NKX6-2, IRF8, RUNX3, FOXG1, HOXD13, MGMT, TWIST1, TTYH3*, and *PRDM2*. To facilitate the cancer-specific comparison, we divided all the samples into three main groups: nasopharyngeal, other malignancies and healthy. We further excluded genes that showed low values across three main groups, resulting in the identification of 9 genes (2 regions from *IRF8*). The methylation levels of these genes are depicted in Fig. 3. All these genes showed significantly elevated methylation levels in cancer samples versus healthy samples. More importantly, many of them (e.g. *HOXD13, IRF8*, and *CCNA1*), also demonstrated notably higher methylation specifically in NPC samples when compared to other malignancies. This finding strongly supports the notion that these candidate genes hold promise as potential biomarkers specific to HNSC.

### psDMR of CPTAC-LSCC reveals potential pan-squamous cell biomarkers

The psDMR analysis conducted on the methylation profiles obtained from 92 paired tumor and normal tissues in CPTAC-LSCC cohort identified a total of 646 significantly differentially methylated regions with FWER < 0.05, with the top regions showing substantial overlap with HNSC results. Notably, 10 genes were consistently identified in the top DMRs across both LSCC and HNSC top 30 DMR list (Table 2), indicating potential pan-squamous cell carcinoma biomarkers: *ALX4, EVX1, HOXD10, MIR311, NKX2-6, SFTA3, ABCC9, MIR409, PCDHGA12*, and *HOXD9. ALX4* is a recognized tumor suppressor that functions in the Wnt/β-catenin signaling pathway and is often silenced through hypermethylation in various cancers. This established biomarker has been validated as differentially methylated in HNSC, colorectal precancerous lesions, and breast cancer ^24^. *EVX1* and *HOXD10* are key developmental genes, playing crucial roles in cellular differentiation and tumor suppression. *NKX2-6* and associated with lung development, have been found to undergo hypermethylation, which may contribute to tumorigenesis in LSCC. *ABCC9* has been linked to drug resistance in cancer, while *MIR311* and *MIR409* are thought to regulate gene expression via hypomethylation, thereby promoting cancer progression. *PCDHGA12*, a member of the protocadherin gene family, has been recognized as a biomarker for lung cancer ^25^. Similarly, *HOXD9*, another member of the HOX gene family, is involved in cell adhesion and developmental processes, further supporting the earlier mention of *HOXD10* and reinforcing their collective importance as epigenetic biomarkers in SCC ^26^ These findings suggest that these genes are strong candidates for further validation and might serve as valuable targets for developing liquid biopsy assays and cfDNA-based diagnostic tools for early detection and monitoring of squamous cell carcinomas.

The HOXA gene family emerged as an important group of biomarkers prominently featured in the top 30 DMRs of LSCC. The hypermethylation of these genes may indicate their epigenetic silencing, further supporting their potential as biomarkers for pan-squamous cell carcinomas. Furthermore, several miRNAs were also identified among the top DMRs, including *MIR411, MIR10B*, and *MIR409*. These miRNAs were hypomethylated, indicating possible regulatory roles in squamous cell carcinoma development through the modulation of gene expression. The presence of miRNAs among the top-ranked DMRs adds another layer of complexity to the epigenetic regulation of these cancers and offers new avenues for exploring miRNA-based biomarkers for diagnosis and prognosis.

Collectively, the top biomarkers identified in both LSCC and HNSC datasets provide a robust set of candidate genes for further exploration. The consistent detection of these DMRs across two independent squamous cell carcinoma datasets demonstrates the reliability and robustness of the psDMR analysis.

## DISCUSSION

Our report addresses a critical issue in the development of liquid biopsy biomarkers for cancer diagnosis and risk stratification, focusing on cfDNA methylation in head and neck cancer. The inclusion of matched normal tissues in our analysis was instrumental in identifying cancer-specific methylation signatures. Our findings challenge the conventional methodology of differential methylation analysis that often overlooks the importance of paired-sample information and fails to address the heterogeneity inherent in HNSC tissue samples. There is prevalent misconception in the filed assuming that fewer samples in DMR analysis will lead to less powerful results. Drawing parallels with the statistical principles of paired versus non-paired statistical tests, the paired-sample approach allowed for a more accurate identification of the tumor-specific epigenetic landscape by filtering out background methylation patterns present in the matched normal tissue. The paired approach holds particular promise for cancers exhibiting substantial heterogeneity, such as head and neck cancer. HNSC arises from the mucosal epithelium of multiple sites such as the oral cavity, larynx, and pharynx. Although they are collectively referred to as HNSC, patients have distinct prognosis and heterogeneous genomic profiles depending on factors such as disease site, human papillomavirus (HPV) status, and history of smoking/tobacco use. Consequently, the DMRs identified through self-controlled psDMR analysis provide a refined list of candidate biomarkers for further validation and clinical testing for highly heterogenous cancers.

One of the critical insights from our analysis is the substantial overlap of hypermethylated DMRs across independent HNSC tumor methylation datasets. This consistency underscores the robustness of the identified regions and supports their relevance for cfDNA methylation biomarker discovery. Notably, the identification and validation of top DMRs within key gene clusters, including HOXD, ZNF, and NKX2, shed new light on their roles in HNSC’s epigenetic landscape, suggesting potential now pathways and mechanisms driving HNSC tumor development and progression.

To the best of our knowledge, this is the first study to conduct a paired sample DMR analysis leveraging the CPTAC-HNSC cohort, and thus our results provide timely insights and valuable resources for future HNSC biomarker study. The processed methylation data of CPTAC-HNSC, based on the improved SeSAMe pipeline, has only been released at GDC Data Portal recently. The utilization of the MethylationEPIC array within the CPTAC cohort allows for the capture of over 850,000 methylation sites, offering significantly more coverage than the 450K array previously employed in the TCGA cohort. This enhanced coverage ensures a more comprehensive scan of the epigenome, facilitating the identification of novel DMRs that may play critical roles in the pathogenesis of HNSC. Moreover, the implementation of CPTAC’s updated protocol for DNA and RNA processing has contributed to a substantial reduction in within-tumor sample heterogeneity. This improvement in sample processing not only refines the quality of the methylation data obtained but also strengthens the reliability of downstream comparative analysis, including those between methylation patterns and gene expression data. As expected, despite a smaller number of paired samples, the CPTAC cohort led to the identification of more DMRs compared to the TCGA cohort. Notably, many of the top identified DMRs have already been recognized as cfDNA or liquid biopsy methylation biomarkers in other types of cancer (as summarized in Table 1). We identified critical genes such as *CALCA, ALX4, BOLL, ZIC1, ADCYAP1, NKX2-6, USP44, HOXD10, EYA4, ZNF577, ZNF781, ZNF154, HOXD9*, and *HOXD10*, underscoring their universal roles as epigenetic markers across various cancers, including HNSC. Additionally, the identification of novel genes with DMRs, such as *PHYHIPL, ABCC9, GFRA1, SALL1, EVX1, HLA-DPB1, SFTA3, RAP1GAP2, KCNN2, ZNF418*, and several microRNAs like *MIR411, MIR409*, and *MIR380*, further highlights the unique epigenetic characteristics of HNSC. All these genes and DMRs are compelling candidates that merit worth further investigation with larger cohort screening trials to confirm their diagnostic and prognostic potential in HNSC.

### Limitations of the study

As mentioned before, the study utilizes a relatively small cohort of paired tumor and normal tissue samples (42 pairs from the CPTAC-HNSC cohort and 50 pairs from the TCGA-HNSC cohort). This limited sample size may reduce the statistical power and generalizability of the findings. However, it is important to highlight that this is already the largest dataset available for HNSC. HNSC is highly heterogeneous, comprising a complex collection of malignancies originating from multiple sites such as the oral cavity, larynx, pharynx, and salivary glands. This diversity results in significant variability influenced by factors such as tumor size, HPV status, and history of smoking. Despite the psDMR analysis accounting for some variability, the intrinsic heterogeneity of HNSC might still pose challenges in identifying universally applicable biomarkers, limiting the study’s applicability across all HNSC subtypes and tumor stages. Finally, the reliance on existing array-based databases for initial methylation region discovery may constrain the scope of the biomarkers. It remains unclear whether the overrepresented hypermethylated sites are due to technical biases or biological factors. This uncertainty highlights the need for further investigation using much larger datasets with paired sample designs to validate and expand upon these findings.

## CONCLUSIONS

In summary, our study utilizing psDMR analysis on the CPTAC-HNSC and TCGA-HNSC datasets successfully identified reliable differentially methylated regions, which could inform more powerful cfDNA methylation biomarker discovery. Within the top DMRs, a significant majority displayed hypermethylation in tumor tissues compared to normal tissues, indicating that the targeted regions identified based on the methylation array (EPIC and 450K) will be relevant to enrichment-based cfDNA methylation methods, which target hypermethylation only. Moreover, many top DMRs were found located in genes that have been previously established as liquid biopsy methylation biomarkers in various cancer types, underscoring their reproducibility and importance as reliable cancer-specific markers. Consistency was also observed between the CPTAC and TCGA in terms of the HOX and ZNF gene families, as well as microRNAs, emphasizing their involvements in the epigenetic landscape of HNSC. Furthermore, we demonstrated the efficacy of targeted region analysis using cfDNA methylation data from OCSCC patients. The analysis identified several significant DMRs in regions previously determined to be significant in the CPTAC analysis. Consistency was also observed with another cfDNA study encompassing multiple cancer types, including NPC. We identified 9 overlapping gens with the DMR genes from the CPTAC analysis. Notably, the methylation levels of many genes not only showed significant elevation in cancer samples compared to healthy samples, but also showed notably higher levels in NPC samples compared to other cancer types, supporting their potential as HNSC-specific biomarkers. Overall, our comprehensive analysis demonstrates the reliability and translational significance of psDMR analysis in HNSC. The findings from the study also provide compelling evidence supporting the utility of DNAme-centric liquid biopsy for precision oncology. Furthermore, the discovered DMRs from our analyiss will provide new insights into HNSCC cancer progression, reveal potential new mechanisms regarding the circulating tumor-immune ecosystem, and may help identify new targets for novel therapeutic treatments.

## Figures and Tables

**Figure 4:**
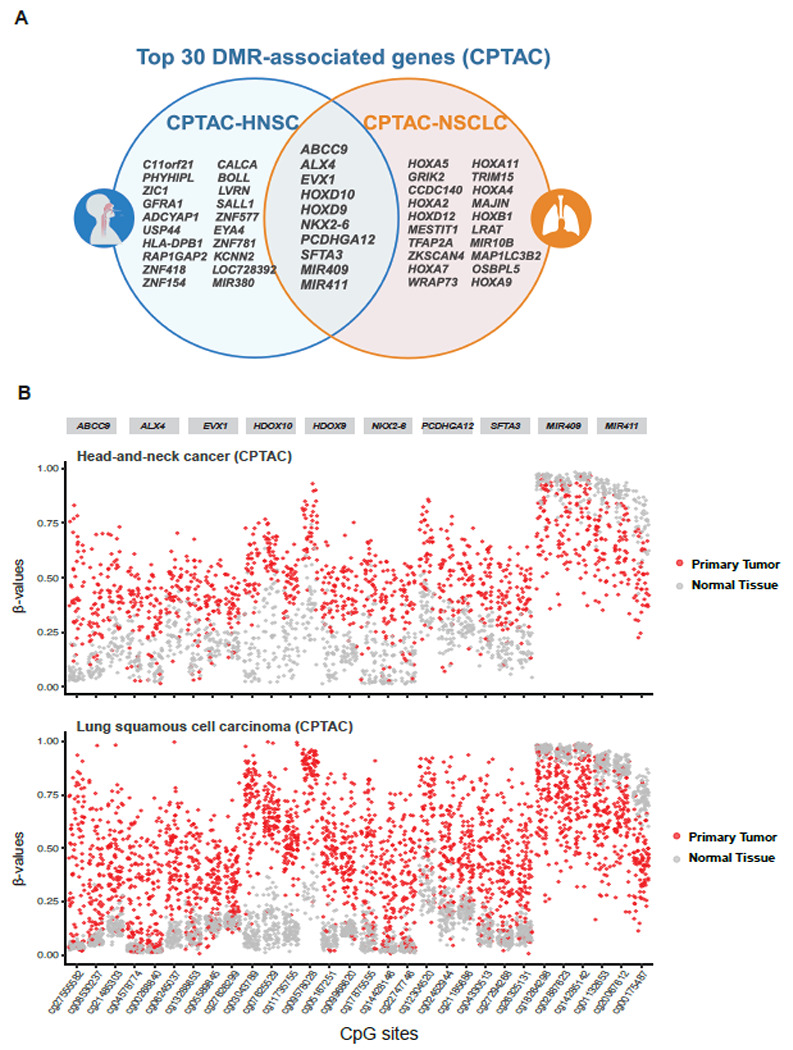
Overlap of DMRs identified from psDMR analysis of CPTAC-HNSC and CPTAC-LSCC datasets reveals potential liquid biopsy biomarkers specific to squamous cell carcinoma. (A) Top 30 overlapped DMR genes identified from HNSC and LSCC. (B) Distribution of DNA methylation levels at top CpG sites across two squamous datasets, comparing tumor and normal tissues.

**Table 1:** Top 30 genomic regions identified using the paired DMR analysis on CPTAC-HNSC data.

DMR (CPTAC)	Chr	Start	End	Gene symbol^†^	EntrezID	No. of CpGs	FWER	FWER.area	DMR state
**1**	chr11	2321770	2323459	**C11orf21**	29125	28	<0.001	0.032	*Hypo*
**2**	chr11	14993378	14995770	**CALCA** ^†^	796	40	<0.001	0.032	Hyper
**3**	chr11	44332385	44333192	**ALX4** ^†^	60529	28	<0.001	0.04	Hyper
**4**	chr10	60936025	60937501	**PHYHIPL**	84457	20	<0.001	0.044	Hyper
**5**	chr12	22093960	22095330	**ABCC9**	10060	16	<0.001	0.044	Hyper
**6**	chr2	198650603	198651590	**BOLL** ^†^	66037	16	<0.001	0.044	Hyper
**7**	chr3	147125712	147127193	**ZIC1** ^†^	7545	23	<0.001	0.044	Hyper
**8**	chr5	115296978	115299088	**LVRN**	206338	19	<0.001	0.048	Hyper
**9**	chr10	118031632	118034031	**GFRA1**	2674	27	<0.001	0.052	Hyper
**10**	chr16	51183988	51185672	**SALL1**	6299	25	<0.001	0.052	Hyper
**11**	chr7	27281216	27282987	**EVX1**	2128	27	<0.001	0.052	Hyper
**12**	chr18	904851	905611	**ADCYAP1** ^†^	116	15	<0.001	0.056	Hyper
**13**	chr19	52390810	52391789	**ZNF577** ^†^	84765	13	<0.001	0.056	Hyper
**14**	chr8	23563572	23565174	**NKX2-6** ^†^	137814	16	<0.001	0.056	Hyper
**15**	chr12	95941571	95943131	**USP44** ^†^	84101	18	<0.001	0.056	Hyper
**16**	chr14	101489415	101490253	**MIR411**	693121	17	<0.001	0.056	*Hypo*
**17**	chr5	140810051	140811520	**PCDHGA12** ^†^	26025	17	<0.001	0.06	Hyper
**18**	chr2	176980673	176981919	**HOXD10** ^†^	3236	12	<0.001	0.064	Hyper
**19**	chr6	133562196	133562776	**EYA4** ^†^	2070	19	<0.001	0.068	Hyper
**20**	chr6	33047944	33048879	**HLA-DPB1**	3115	17	<0.001	0.076	Hyper
**21**	chr14	36982447	36983791	**SFTA3**	253970	15	<0.001	0.076	Hyper
**22**	chr19	38182773	38183460	**ZNF781** ^†^	163115	12	<0.001	0.076	Hyper
**23**	chr17	2699249	2700093	**RAP1GAP2**	23108	12	<0.001	0.076	*Hypo*
**24**	chr5	113697330	113698847	**KCNN2**	3781	17	<0.001	0.076	Hyper
**25**	chr2	176986535	176987605	**HOXD9** ^†^	3235	12	<0.001	0.076	Hyper
**26**	chr19	58446312	58446988	**ZNF418**	147686	11	<0.001	0.076	Hyper
**27**	chr14	101531403	101532352	**MIR409**	574413	18	<0.001	0.076	*Hypo*
**28**	chr17	5402883	5404581	**LOC728392**	728392	16	<0.001	0.076	Hyper
**29**	chr19	58220080	58220955	**ZNF154** ^†^	7710	11	<0.001	0.076	Hyper
**30**	chr14	101491151	101492406	**MIR380**	494329	14	<0.001	0.076	*Hypo*

† Genes that have been previously established as cfDNA or liquid biopsy methylation biomarkers in at least one cancer type.

**Table 2:** Top 30 genomic regions identified using the paired DMR analysis on CPTAC-LSCC data.

DMR (TCGA)	Chr	Start	End	Gene Symbol^†^*	EntrezID	No. of CpGs	FWER	FWER.area	DMR state
**1**	chr7	27183133	27184821	**HOXA5** ^†^	3202	33	<0.001	0.004	Hyper
**2**	chr7	27224700	27226329	**HOXA11** ^†^	3207	29	<0.001	0.008	Hyper
**3**	chr11	44332454	44333192	**ALX4** ^†^ *	60529	27	<0.001	0.024	Hyper
**4**	chr7	27280914	27282987	**EVX1** *	2128	29	<0.001	0.028	Hyper
**5**	chr6	101846603	101847656	**GRIK2** ^†^	2898	17	<0.001	0.028	Hyper
**6**	chr6	30130881	30132715	**TRIM15**	89870	26	<0.001	0.028	Hypo
**7**	chr2	223162587	223163809	**CCDC140**	151278	17	<0.001	0.028	Hyper
**8**	chr7	27168609	27171528	**HOXA4** ^†^	3201	31	<0.001	0.028	Hyper
**9**	chr7	27142200	27143806	**HOXA2**	3199	21	<0.001	0.028	Hyper
**10**	chr11	64739253	64739852	**MAJIN**	283129	14	<0.001	0.028	Hyper
**11**	chr2	176980673	176981469	**HOXD10** ^†^ *	3236	10	<0.001	0.032	Hyper
12	chr2	176963948	176964720	**HOXD12**	3238	11	<0.001	0.032	Hyper
**13**	chr17	46607828	46608570	**HOXB1**	3211	13	<0.001	0.032	Hyper
**14**	chr14	101489415	101490253	**MIR411** *	693121	17	<0.001	0.036	Hypo
**15**	chr7	130129946	130131484	**MESTIT1**	317751	25	<0.001	0.036	Hyper
**16**	chr4	155661691	155664242	**LRAT**	9227	19	<0.001	0.04	Hyper
**17**	chr8	23563572	23565174	**NKX2-6** ^†^ *	137814	16	<0.001	0.04	Hyper
**18**	chr14	36982447	36984132	**SFTA3** *	253970	16	<0.001	0.04	Hyper
**19**	chr6	10421460	10422874	**TFAP2A** ^†^	7020	10	<0.001	0.04	Hyper
**20**	chr2	177014555	177015125	**MIR10B**	406903	11	<0.001	0.04	Hyper
**21**	chr12	22093960	22095330	**ABCC9** *	10060	15	<0.001	0.04	Hyper
**22**	chr14	101531403	101532352	**MIR409** *	574413	18	<0.001	0.044	Hypo
**23**	chr6	28226885	28227482	**ZKSCAN4**	387032	13	<0.001	0.044	Hyper
**24**	chr12	116996359	116997185	**MAP1LC3B2**	643246	11	<0.001	0.044	Hypo
**25**	chr5	140810051	140811343	**PCDHGA12** *	26025	16	<0.001	0.044	Hyper
**26**	chr7	27195602	27196825	**HOXA7** ^†^	3204	16	<0.001	0.044	Hyper
**27**	chr2	176986535	176987605	**HOXD9** ^†^ *	3235	11	<0.001	0.044	Hyper
**28**	chr11	3187362	3188016	**OSBPL5**	114879	19	<0.001	0.044	Hypo
**29**	chr1	3567163	3568245	**WRAP73**	49856	19	<0.001	0.044	Hyper
**30**	chr7	27204663	27205658	**HOXA9**	3205	11	<0.001	0.044	Hyper

† Genes that have been previously established as cfDNA or liquid biopsy methylation biomarkers in at least one cancer type.

* Genes identified among the top 30 DMRs in the CPTAC-HNSC data analysis, indicating strong potential as specific biomarkers for squamous cell carcinoma.

## Data Availability

All data used in the analysis are available on the **CPTAC platform**.

## References

[1] Shen SY, Sensitive tumour detection and classification using plasma cell-free DNA methylomes. Nature. 2018;563:579–83. 30429608 10.1038/s41586-018-0703-0

[2] Jin S , Efficient detection and post-surgical monitoring of colon cancer with a multi-marker DNA methylation liquid biopsy. Proceedings of the National Academy of Sciences 118, e2017421118 (2021). 10.1073/pnas.2017421118 PMC7865146 33495330

[3] Zhou X, Lu X, Wu H, Liu J, Huang H. Diagnostic performance of SHOX2 promoter methylation as biomarker for lung cancer identification: A meta-analysis update. Thorac Cancer. 2021;12:3327–32. 34741433 10.1111/1759-7714.14206 PMC8671898

[4] Kneip C, SHOX2 DNA methylation is a biomarker for the diagnosis of lung cancer in plasma. J Thorac Oncol. 2011;6:1632–8. 21694641 10.1097/JTO.0b013e318220ef9a

[5] Bergheim J, Potential of quantitative SEPT9 and SHOX2 methylation in plasmatic circulating cell-free DNA as auxiliary staging parameter in colorectal cancer: a prospective observational cohort study. Br J Cancer. 2018;118:1217–28. 29610456 10.1038/s41416-018-0035-8 PMC5943265

[6] Noushmehr H, Herrgott G, Morosini NS, Castro AV. Noninvasive approaches to detect methylation-based markers to monitor gliomas. Neuro-Oncology Adv. 2022;4:ii22–32. 10.1093/noajnl/vdac021 PMC9650474 36380867

[7] Wei B, A panel of DNA methylation biomarkers for detection and improving diagnostic efficiency of lung cancer. Sci Rep. 2021;11:16782. 34408226 10.1038/s41598-021-96242-6 PMC8373977

[8] van Zogchel LM, Novel circulating hypermethylated RASSF1A ddPCR for liquid biopsies in patients with pediatric solid tumors. JCO Precision Oncol. 2021;5:1738–48. 10.1200/PO.21.00130 PMC8608265 34820594

[9] Chen S, Duan H, Zhang D, Sun G. Correlation between RASSF1A Methylation in Cell-Free DNA and the Prognosis of Cancer Patients: A Systematic Review and Meta-Analysis. J Oncol 2022(2022). 10.1155/2022/3458420 PMC9071870 35528240

[10] Boldrin E, Detection of LINE-1 hypomethylation in cfDNA of Esophageal Adenocarcinoma Patients. Int J Mol Sci. 2020;21:1547. 32102481 10.3390/ijms21041547 PMC7073170

[11] Nagai Y, LINE-1 hypomethylation status of circulating cell-free DNA in plasma as a biomarker for colorectal cancer. Oncotarget. 2017;8:11906. 28060757 10.18632/oncotarget.14439 PMC5355314

[12] Liang W Accurate diagnosis of pulmonary nodules using a noninvasive DNA methylation test. J Clin Investig 131(2021). 10.1172/JCI145973 PMC8121527 33793424

[13] Patel KB, Plasma cell-free DNA methylome profiling in pre-and post-surgery oral cavity squamous cell carcinoma. Mol Carcinog. 2023;62:493–502. 36636912 10.1002/mc.23501 PMC10023468

[14] Zhou W, Triche TJ Jr, Laird PW, Shen H. SeSAMe: reducing artifactual detection of DNA methylation by Infinium BeadChips in genomic deletions. Nucleic Acids Res. 2018;46:e123–123. 30085201 10.1093/nar/gky691 PMC6237738

[15] Huang C, Proteogenomic insights into the biology and treatment of HPV-negative head and neck squamous cell carcinoma. Cancer Cell. 2021;39:361–79. e316. 33417831 10.1016/j.ccell.2020.12.007 PMC7946781

[16] Loyo M, A survey of methylated candidate tumor suppressor genes in nasopharyngeal carcinoma. Int J Cancer. 2011;128:1393–403. 20473931 10.1002/ijc.25443 PMC2955155

[17] Hu S, Detection of serum deoxyribonucleic acid methylation markers: a novel diagnostic tool for thyroid cancer. J Clin Endocrinol Metabolism. 2006;91:98–104. 10.1210/jc.2005-1810 16263813

[18] Liggett TE, Distinctive DNA methylation patterns of cell-free plasma DNA in women with malignant ovarian tumors. Gynecol Oncol. 2011;120:113–20. 21056906 10.1016/j.ygyno.2010.09.019 PMC3004216

[19] da Silva Martinelli CM, MGMT and CALCA promoter methylation are associated with poor prognosis in testicular germ cell tumor patients. Oncotarget. 2017;8:50608. 28881587 10.18632/oncotarget.11167 PMC5584175

[20] Paluszczak J, Prognostic significance of the methylation of Wnt pathway antagonists—CXXC4, DACT2, and the inhibitors of sonic hedgehog signaling—ZIC1, ZIC4, and HHIP in head and neck squamous cell carcinomas. Clin Oral Invest. 2017;21:1777–88. 10.1007/s00784-016-1946-5 PMC5442212 27553089

[21] Yang J, ALX4, an epigenetically down regulated tumor suppressor, inhibits breast cancer progression by interfering Wnt/β-catenin pathway. J Experimental Clin Cancer Res. 2017;36:1–15. 10.1186/s13046-017-0643-9 PMC5706407 29183346

[22] Paço A, de Bessa Garcia SA, Freitas R. Methylation in HOX clusters and its applications in cancer therapy. Cells. 2020;9:1613. 32635388 10.3390/cells9071613 PMC7408435

[23] Gaykalova DA, Outlier analysis defines zinc finger gene family DNA methylation in tumors and saliva of head and neck cancer patients. PLoS ONE. 2015;10:e0142148. 26544568 10.1371/journal.pone.0142148 PMC4636259

[24] Lleras RA, Unique DNA methylation loci distinguish anatomic site and HPV status in head and neck squamous cell carcinoma. Clin Cancer Res. 2013;19:5444–55. 23894057 10.1158/1078-0432.CCR-12-3280 PMC3892374

[25] Jeong IB, PCDHGA12 methylation biomarker in bronchial washing specimens as an adjunctive diagnostic tool to bronchoscopy in lung cancer. Oncol Lett. 2018;16:1039–45. 29963180 10.3892/ol.2018.8699 PMC6019926

[26] Rodrigues MF, Esteves CM, Xavier FC, Nunes FD. Methylation status of homeobox genes in common human cancers. Genomics. 2016;108:185–93. 27826049 10.1016/j.ygeno.2016.11.001

